# Supplementary material for: Associations between the VDR Gene rs731236 (TaqI) Polymorphism and Bone Mineral Density in Postmenopausal Women from the RAC-OST-POL
Source: Biomedicines. 2024 Apr 20;12(4):917. doi: 10.3390/biomedicines12040917 (PMC11048207; doi:10.3390/biomedicines12040917)
Supplement: Supplementary file 1 [file biomedicines-12-00917-s001.zip › biomedicines-2937091-supplementary.pdf]

Table S1. *VDR* gene variations information.

**SNP**	**Ref**	**Alt**	**Position**	**GMAF**
rs7975232 ApaI	C	A	chr12:47845054	0.4959
rs731236 TaqI	A	G	chr12:47844974	0.264
rs1544410 Bsm I	C	T	chr12:47846052	0.2649

SNP - single nucleotide variations
Ref - referance allele
Alt - alternative allele
Position - position in the genome according to Homo sapiens (human) genome assembly GRCh38 (hg38_HC) GRCh38.p14 chr 12
GMAF – global minor allele frequency
